# Prospects for daily online adaptive radiotherapy for cervical cancer: Auto-contouring evaluation and dosimetric outcomes

**DOI:** 10.1186/s13014-024-02398-6

**Published:** 2024-01-12

**Authors:** Yu Zhang, Guangyu Wang, Yankui Chang, Zhiqun Wang, Xiansong Sun, Yuliang Sun, Zheng Zeng, Yining Chen, Ke Hu, Jie Qiu, Junfang Yan, Fuquan Zhang

**Affiliations:** 1 Department of Radiation Oncology, Peking Union Medical College Hospital, Chinese Academy of Medical Science and Peking Union Medical College, Beijing, 100730 China; 2 Department of Radiation Oncology, State Key Laboratory of Complex Severe and Rare Diseases, Peking Union Medical College Hospital, Chinese Academy of Medical Science and Peking Union Medical College, Beijing, China; 3 School of Nuclear Science and Technology, University of Science and Technology of China, Hefei, China

**Keywords:** Online adaptive radiotherapy, Cone-beam CT, Cervical neoplasm, Auto-contouring

## Abstract

**Background:**

Training senior radiation therapists as “adapters” to manage influencers and target editing is critical in daily online adaptive radiotherapy (oART) for cervical cancer. The purpose of this study was to evaluate the accuracy and dosimetric outcomes of automatic contouring and identify the key areas for modification.

**Methods:**

A total of 125 oART fractions from five postoperative cervical cancer patients and 140 oART fractions from five uterine cervical cancer patients treated with daily iCBCT-guided oART were enrolled in this prospective study. The same adaptive treatments were replanned using the Ethos automatic contours workflow without manual contouring edits. The clinical target volume (CTV) was subdivided into several separate regions, and the average surface distance dice (ASD), centroid deviation, dice similarity coefficient (DSC), and 95% Hausdorff distance (95% HD) were used to evaluate contouring for the above portions. Dosimetric results from automatic oART plans were compared to supervised oART plans to evaluate target volumes and organs at risk (OARs) dose changes.

**Results:**

Overall, the paired CTV had high overlap rates, with an average DSC value greater than 0.75. The uterus had the largest consistency differences, with ASD, centroid deviation, and 95% HD being 2.67 ± 1.79 mm, 17.17 ± 12 mm, and 10.45 ± 5.68 mm, respectively. The consistency differences of the lower nodal CTV_left_ and nodal CTV_right_ were relatively large, with ASD, centroid deviation, and 95% HD being 0.59 ± 0.53 mm, 3.6 ± 2.67 mm, and 5.41 ± 4.08 mm, and 0.59 ± 0.51 mm, 3.6 ± 2.54 mm, and 4.7 ± 1.57 mm, respectively. The automatic online-adapted plan met the clinical requirements of dosimetric coverage for the target volume and improved the OAR dosimetry.

**Conclusions:**

The accuracy of automatic contouring from the Ethos adaptive platform is considered clinically acceptable for cervical cancer, and the uterus, upper vaginal cuff, and lower nodal CTV are the areas that need to be focused on in training.

## Introduction

Cervical cancer is one of the most common malignant tumours, ranking first in the incidence of gynaecological malignancies [1, 2]. Radiotherapy plays a crucial role in cervical cancer. For uterine cervical cancer, the treatment outcomes after radiotherapy and surgery for early-stage cases are comparable [3], and chemoradiotherapy is the mainstay of treatment in locally advanced diseases [4, 5]. For patients with postoperative high-risk factors, postoperative radiotherapy can reduce local recurrence [6, 7]. However, the amount of unpredictable organ motion, bladder-rectum filling, and tumour regression all have implications for precision radiotherapy [8–11], influencing the precision of radiotherapy and requiring large planning target volume (PTV) margins to account for these complex intrapelvic organ dynamics. The treatment volumes with large PTV margins contained a mass of organs at risk (OARs), leading to adverse responses to treatment, such as urinary and gastrointestinal complications [12, 13].

Online adaptive radiotherapy (oART), which can involve conducting full reoptimization of the anatomy of the day, has been demonstrated to be feasible within a clinical setting [14]. Among the approaches, iterative cone-beam computed tomography (iCBCT)-guided oART could significantly shorten the total time for patients to maintain a fixed position with good pelvic soft tissue display resolution and shows an enormous advantage in cervical cancer [15]. Ethos (Varian Medical Systems/Siemens Healthineers, Palo Alto, CA) could automatically segment the target and normal tissue structure and reoptimize the treatment plan, offering iCBCT-guided oART. However, the clinical use of oART has been limited due to cumbersome redelineation, which requires physicians to be on-site for daily adjustments. Currently, some institutions design senior radiation therapist training delineation courses as “adapters” to manage the oART workflow in general, including adjusting daily delineations [16]. Manual contouring is usually the most time-consuming and error-prone part of oART [14]; thus, identifying the degree of editing of different regions may be important for training adapters. Herein, we assessed the accuracy of daily automatic contouring and dosimetric outcomes for cervical cancer with or without uterus to explore the benefit of improving the training adapter with iCBCT-guided oART.

## Materials and methods

### Patients

Between December 2022 and June 2023, 125 oART fractions from five postoperative cervical cancer patients and 140 oART fractions from five uterine cervical cancer patients treated with daily iCBCT-guided oART (Ethos Linac) were enrolled in this prospective study. All patients were placed in the supine position and fixed with thermoplastic film during positioning. Postoperative patients had indications for adjuvant pelvic radiotherapy and received 45 Gy in 1.8 Gy daily fraction to PTV, while patients with radical radiotherapy received 50.4 Gy in 28 fractions.

### Pre-implementation treatment planning

The clinical target volume (CTV) contouring was performed according to the Radiation Therapy Oncology Group (RTOG) consensus guidelines [17, 18]. The CTV of postoperative treatment of cervical cancer included separate nodal CTV (CTV-N) and vaginal CTV (CTV-V) contours. The CTV was expanded by a uniform three-dimensional planning margin of 5 mm to generate the PTV. The CTV for cervical cancer with an intact cervix consisted of CTV-N, uterus (CTV-U), parametria, cervix (compassed gross tumour volume) and vaginal tissues (CTV-C). A uniform 5-mm CTV-to-PTV margin was used for CTV-C and CTV-N, and a 10 mm margin was used to cover more variable CTV-U. The OARs included the bladder, bowel, rectum, bilateral femoral heads, bone marrow, and spinal cord.

A template with a departmental prioritized list of clinical goals is required for plan optimization and evaluation. Each goal consists of a minimum acceptable value and an ideal value. In order to achieve desirable dose distribution, the clinical goal was added as the minimum acceptable value, and an optimization objective was added as the ideal value. The treatment plans were calculated using intensity-modulated radiotherapy (IMRT) with 9 laterally equidistant fields (Gantry angles: 180, 140, 100, 60, 20, 340, 300, 260 and 220°) and a preview of the dose distribution was generated by the system. Based on the dose preview, the clinical goals that were used as optimization objectives were adjusted to further improve the plan. Subsequently, the final reference plan was selected and approved by the physicians.

### Daily adaptive workflow

All patients underwent a rigorous bladder-rectal preparation, emptying their bladder and rectum one hour and forty minutes before the appointment, followed by an intake of 450 or 500 ml water within 10 min according to their height and weight before simulation and per setup, thus guaranteeing the daily repetition of the treatment. After the first iCBCT scan was performed, the “influencer” structures (bladder, rectum and bowel) were automatically contoured using convolutional neural network-based auto-segmentation model on daily iCBCT images. Then, the elastic deformation vector fields were used to propagate contours of noninfluencer OARs from planning CT to daily iCBCT, and the contours of influencers were used to generate structures-guided registration deformation vector fields for propagating CTV from the planning CT to the daily iCBCT. After the physician reviewed and manually modified the above structures, a newly optimized treatment plan was generated using the beam setup and clinical goals of the reference plan. Simultaneously, the other plan that calculated the dose distribution of the unaltered reference plan based on maximized PTV coverage, where using isocenter translation to align the PTV on reference plan CT with those propagated toward the iCBCT. The newly created plan was called the adapted plan, while the reference plan recalculated on current anatomy was called the scheduled plan. Treatment was delivered after plan selection and position verification by a second iCBCT scan. The modifying degree of structures was referred to as the method applied by Byrne et al. [14] and were classified by physicians as either no edits (no change to the structure), minor edits (no more than 10% of slices need small changes), moderate edits (more than 10% of the slices need small changes, or no more than 10% of the slices need big changes major revisions), or major edits (big changes that do not include minor and moderated changes, or structural deletions and recontours).

### Automatic contour and planning

The daily first iCBCT scans were uploaded to the Ethos oART emulator, which had the same software and functionality as the clinical version, for daily adaptive replanning, and two reference plans based on the same dosimetric constraint planning templates as the reference CT were generated. The contours of influencers and CTVs were generated using the fully automatic workflow in Ethos without manual corrections.

### Contour and dosimetry comparison

**Fig. 1:**
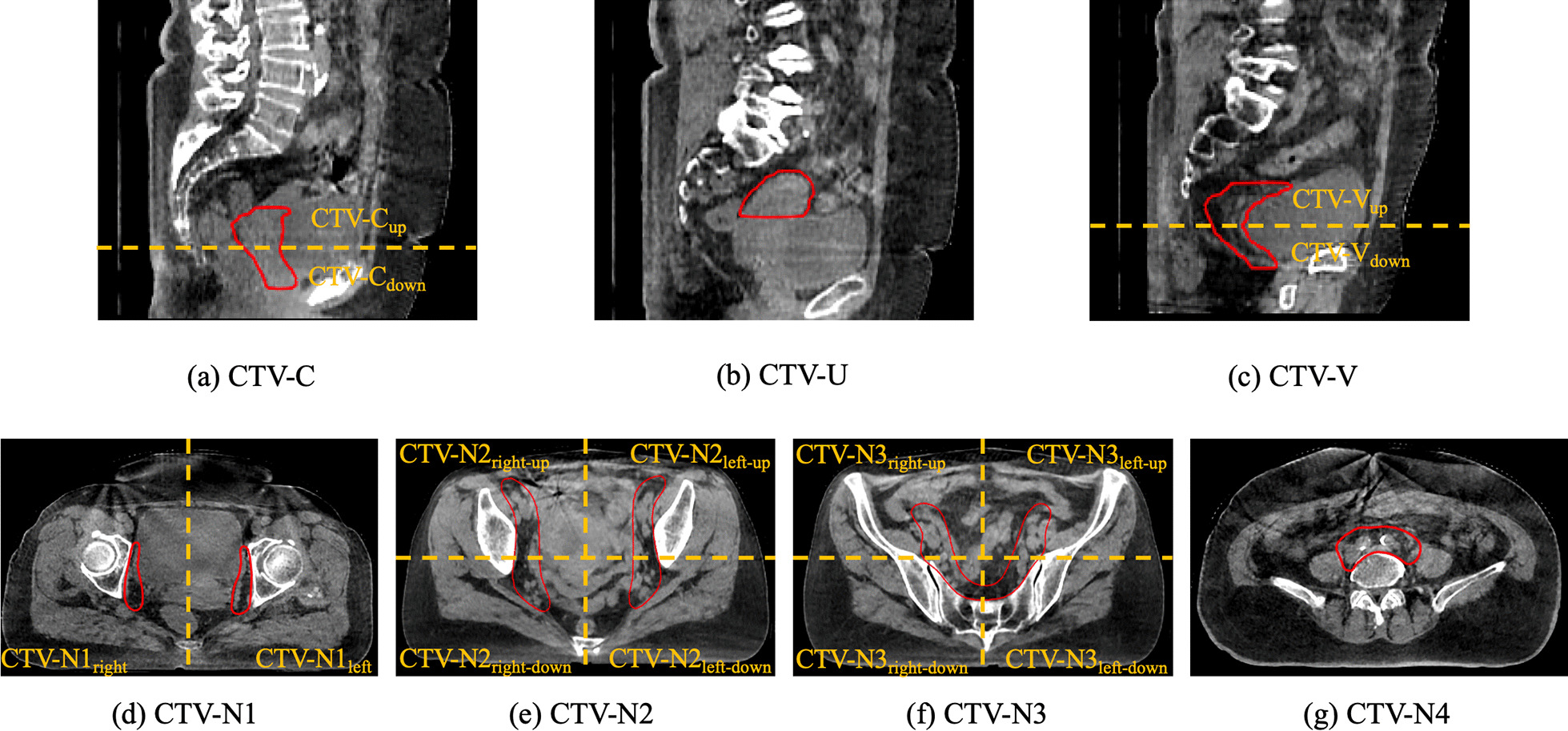
The subdivision of CTV. **a** The CTV-C of the uterine cervical cancer was evenly divided into CTV-C_up_ and CTV-C_down_ according to the length of the target. **b** The uterus of the uterine cervical cancer (CTV-U). (**c**)The CTV-V of the postoperative cervical cancer was evenly divided into CTV-V_up_ and CTV-V_down_ according to the length of the target. **d**–**g** The CTV-N was firstly divided into four portions from supper to low, and the boundaries were the bifurcation of the common iliac artery, the appearance of the piriformis muscle, and the appearance of the femoral head. Then, the middle two portions were subdivided into four sections along the transverse axis and midline of the body, and the lowest portion was divided into two parts according to the midline of the body

To evaluate the agreement between auto-contouring and manually corrected contouring, the targets were segmented into several parts. The CTV-N was first divided into four portions from supper to low, and the boundaries were the bifurcation of the common iliac artery, the appearance of the piriformis muscle, and the appearance of the femoral head. Then, the middle two portions were subdivided into four sections along the transverse axis and midline of the body, and the lowest portion was divided into 2 parts according to the midline of the body. Thus, CTV-N was subdivided into 11 parts; an illustration of the subdivision is shown in Fig. 1. The CTV-V of the postoperative radiotherapy and the CTV-C of the radical radiotherapy were divided into supper and lower portions according to the length of the target.

The average surface distance dice (ASD), centroid deviation, dice similarity coefficient (DSC), and 95% Hausdorff distance (95% HD) were used to evaluate contouring for the above portions. ASD is used to measure the relative distance between two surfaces [19], DSC is used to measure the similarity of two sets [20], and 95% HD is used to measure the degree of boundary coincidence [21]. Centroid deviation (CD) was defined as, ${\text{CD}}\left({\text{A}},{\text{B}}\right)=\sqrt{{\left({{\text{X}}}_{{\text{A}}}-{{\text{X}}}_{{\text{B}}}\right)}^{2}+{\left({{\text{Y}}}_{{\text{A}}}-{{\text{Y}}}_{{\text{B}}}\right)}^{2}+{\left({{\text{Z}}}_{{\text{A}}}-{{\text{Z}}}_{{\text{B}}}\right)}^{2}}$, where (X_A_,Y_A_,Z_A_) and (X_B_,Y_B_,Z_B_) were the centroids of A and B, respectively.

The volume of CTV and PTV receiving 100% of the prescribed dose (V_100%_) and the dose to OARs were recorded for each fraction and compared between the adapted plan and scheduled plan. The scheduled and adapted plan from A-ART (Automatic ART, contouring was generated using the fully automatic workflow in Ethos) and S-ART (Supervised ART, contouring was generated by manual corrections by physicians) were compared. The dosimetric outcomes of A-ART were re-calculated with manually edited contours which was same as S-ART. The consensus guidelines for CTV-N delineation for postoperative and uterine cervical cancer are the same; thus, the results of the evaluation are combined for contour and dosimetry comparison. The comparisons among dose results were analysed by *t* test. *p* values < 0.05 denoted a significant difference.

## Results

### Timing data and contouring accuracy

For the whole clinical process of oART, the average total adaptive time for A-ART postoperative cervical cancer and A-ART uterine cervical cancer from first iCBCT acquisition to plan selection were 7 min 46 s (range 6 min 47 s to 9 min 22 s) and 8 min 29 s (range 7 min 11 s to 10 min 03 s), while the average time for S-ART postoperative cervical cancer and S-ART uterine cervical cancer were 14 min 32 s (range 10 min 35 s to 20 min 35 s) and 17 min 55 s (range 11 min 37 s to 28 min 33 s).

**Fig. 2:**
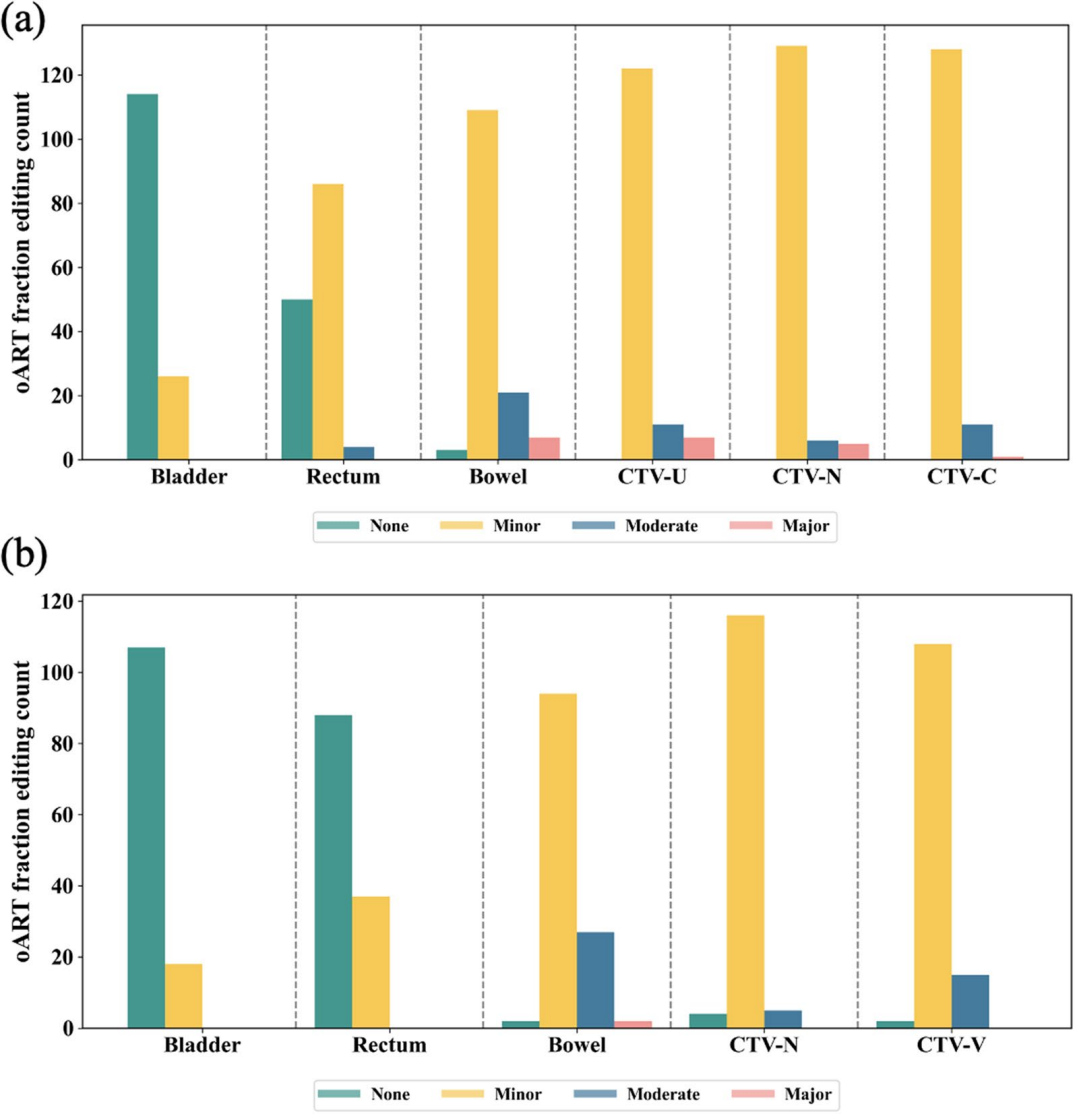
Frequency of edits needed for influencer structures (bladder, rectum and bowel) and CTV (CTV-N, CTV-V, CTV-U and CTV-C) in uterine cervical cancer (**a**) and postoperative cervical cancer (**b**)

For postoperative cervical cancer, each fraction consisted of 3 editors for the influencer structures (bladder, rectum and bowel), 2 editors for CTV (CTV-N and CTV-V), and 3 editors for the influencer structures and 3 editors for CTV (CTV-U, CTV-C and CTV-N) for the intact cervix. Overall, for postoperative cervical cancer, 92.3% (346/375 times) of the influencers and 92% (230/250 times) of CTV needed no or minor edits. In addition, 92.1% (387/420 times) of the influencers and 91.4% (384/420 times) of CTV needed no or minor edits for cervical cancer treated by radical oART. Figure 2 shows the frequency of CTV and influencer editing needed.

### Contouring comparing

The comparison between A-ART and S-ART contouring of postoperative and uterine cervical cancer is given in Table 1. Overall, the paired CTV had high overlap rates, with DSC values greater than 0.75, and CTV-U had the largest consistency differences, with ASD, centroid deviation, DSC, and 95% HD being 2.67 ± 1.79 mm, 17.17 ± 12 mm, 0.76 ± 0.12, and 10.45 ± 5.68, respectively. For CTV-V in the postoperative group and CTV-C in the radical group, the difference in the upper region was significantly greater than that in the lower region in the ASD, DSC and 95% HD. On average, for 265 paired CTV-N, the consistency differences of CTV-N1_left_ and CTV-N1_right_ were relatively large, with ASD, centroid deviation, DSC, and 95% HD being 0.59 ± 0.53 mm, 3.6 ± 2.67 mm, and 5.41 ± 4.08 mm and 0.59 ± 0.51 mm, 3.6 ± 2.54 mm, and 4.7 ± 1.57 mm, respectively.

**Table 1:** Comparison between A-ART and S-ART contouring of postoperative and uterine cervical cancer

Region	ASD (mm)	Centroid deviation (mm)	DSC	95% HD (mm)
CTV-N1_left_	0.59 ± 0.53	3.6 ± 2.67	0.91 ± 0.05	5.41 ± 4.08
CTV-N1_right_	0.59 ± 0.51	3.6 ± 2.54	0.86 ± 0.06	4.7 ± 1.57
CTV-N2_left-up_	0.32 ± 0.34	2.71 ± 2.79	0.95 ± 0.05	2.25 ± 1.77
CTV-N2_right-up_	0.24 ± 0.33	2.21 ± 2.4	0.96 ± 0.04	1.72 ± 2.06
CTV-N2_left-down_	0.37 ± 0.42	4.43 ± 4.89	0.95 ± 0.04	3.45 ± 4.42
CTV-N2_right-down_	0.35 ± 0.4	3.9 ± 4.08	0.96 ± 0.03	3.26 ± 4.28
CTV-N3_left-up_	0.32 ± 0.31	4.67 ± 3.93	0.95 ± 0.04	2.04 ± 1.34
CTV-N3_right-up_	0.41 ± 0.4	5.28 ± 5.08	0.94 ± 0.05	2.51 ± 1.79
CTV-N3_left-down_	0.32 ± 0.34	3.53 ± 2.75	0.91 ± 0.07	2.29 ± 1.64
CTV-N3_right-down_	0.33 ± 0.42	4.13 ± 4.23	0.91 ± 0.09	2.29 ± 1.96
CTV-N4	0.59 ± 0.54	2.4 ± 2.27	0.93 ± 0.05	3.62 ± 2.93
CTV-V_down_	1.13 ± 0.92	5.21 ± 3.73	0.84 ± 0.09	4.61 ± 2.05
CTV-V_up_	1.84 ± 0.84	8.9 ± 8.82	0.79 ± 0.07	7.3 ± 3.31
CTV-C_down_	0.66 ± 0.42	4.31 ± 3.21	0.91 ± 0.05	3.69 ± 1.6
CTV-C_up_	1.46 ± 0.74	7.93 ± 5.13	0.86 ± 0.06	6.89 ± 3.26
CTV-U	2.67 ± 1.79	17.17 ± 12	0.76 ± 0.12	10.45 ± 5.68
CTV-V	1.75 ± 0.88	6.03 ± 5.07	0.81 ± 0.07	6.9 ± 2.88
CTV-C	1.31 ± 0.62	5.25 ± 3.06	0.88 ± 0.05	6.06 ± 2.65
CTV-N	0.53 ± 0.39	2.55 ± 1.86	0.94 ± 0.04	3.17 ± 1.28

*ASD* Average surface distance dice; *DSC* The dice similarity coefficient; *95% HD* 95% Hausdorff distance

### Dosimetric outcomes

Tables 2 and 3 lists results of dosimetric outcomes for 125 and 140 oART fractions of postoperative and uterine cervical cancer based on manually corrected contours. For postoperative cervical cancer, the adapted plan achieved superior dosimetric coverage for the target volume compared to the scheduled plan, with V_100%_ of CTV-N (99.94% ± 0.08%) and CTV-V (99.98% ± 0.04%). The advantage was more significant on PTV, especially on PTV-V, which is greatly affected by bladder and rectal filling, and the V_100%_ of the S-Scheduled plan was 90.96% ± 5.78%, respectively. Compared with the S- Scheduled plan over all sessions, the S-Adapted plan could significantly improve the OAR dosimetry from high dose coverage to low dose coverage, including in the bladder, rectum and bowel. Although the A-Adapted plan met the clinical requirements, it was inferior to the S-Adapted plan. Dosimetric outcomes of uterine cervical cancer were similar.

**Table 2:** Dosimetric outcomes for 125 oART fraction of postoperative cervical cancer

ROIs	Dosimetric metrics	A-Adapted plan	A-Scheduled plan	S-Adapted plan	S-Scheduled plan	*p*1 value	*p*2 value
CTV-N	V_100%_ (%)	99.20 ± 0.92	91.37 ± 8.54	99.94 ± 0.08	97.19 ± 4.57	< 0.05	< 0.05
CTV-V	V_100%_ (%)	97.13 ± 2.99	94.73 ± 4.23	99.98 ± 0.04	96.85 ± 3.70	< 0.05	< 0.05
PTV-N	V_100%_ (%)	91.92 ± 2.32	85.11 ± 8.39	96.75 ± 0.32	91.84 ± 4.72	< 0.05	< 0.05
PTV-V	V_100%_ (%)	91.52 ± 4.36	87.95 ± 5.74	96.41 ± 0.87	90.96 ± 5.78	< 0.05	< 0.05
Bladder	V_40Gy_ (%)	23.12 ± 3.53	27.65 ± 5.38	24.05 ± 2.25	29.09 ± 5.25	< 0.05	= 0.04
	V_30Gy_ (%)	36.98 ± 3.76	41.50 ± 4.99	37.87 ± 3.13	43.73 ± 6.10	< 0.05	= 0.05
	V_20Gy_ (%)	56.44 ± 4.92	62.49 ± 7.30	59.15 ± 7.02	65.46 ± 9.99	< 0.05	< 0.05
	V_10Gy_ (%)	91.10 ± 3.20	93.96 ± 3.23	93.07 ± 3.42	94.52 ± 3.36	< 0.05	< 0.05
	D_mean_ (Gy)	0.81 ± 0.07	0.90 ± 0.10	0.84 ± 0.07	0.94 ± 0.11	< 0.05	< 0.05
	D_50%_ (Gy)	0.92 ± 0.05	0.98 ± 0.07	0.95 ± 0.05	1.01 ± 0.08	< 0.05	< 0.05
Rectum	V_40Gy_ (%)	39.99 ± 17.09	43.03 ± 19.91	37.50 ± 9.46	38.47 ± 16.99	= 0.58	= 0.38
	V_30Gy_ (%)	59.72 ± 16.57	66.97 ± 20.24	57.01 ± 9.97	62.06 ± 16.47	< 0.05	= 0.23
	V_20Gy_ (%)	77.07 ± 12.88	82.49 ± 14.81	74.91 ± 8.13	80.48 ± 13.00	< 0.05	= 0.28
	V_10Gy_ (%)	92.22 ± 6.33	93.75 ± 5.51	91.70 ± 5.79	93.15 ± 5.25	= 0.04	= 0.76
	D_mean_ (Gy)	1.23 ± 0.28	1.31 ± 0.28	1.19 ± 0.17	1.26 ± 0.25	= 0.02	= 0.39
	D_50%_ (Gy)	1.14 ± 0.18	1.20 ± 0.21	1.12 ± 0.12	1.16 ± 0.18	= 0.01	= 0.44
Bone Marrow	V_10Gy_ (%)	83.14 ± 4.51	85.15 ± 4.56	82.78 ± 5.00	84.73 ± 4.28	< 0.05	= 0.99
	D_90%_ (Gy)	0.25 ± 0.07	0.28 ± 0.06	0.25 ± 0.07	0.27 ± 0.06	< 0.05	= 0.86
	D_mean_ (Gy)	0.79 ± 0.03	0.82 ± 0.03	0.79 ± 0.03	0.81 ± 0.03	< 0.05	= 0.43
Femur head left	V_30Gy_ (%)	1.47 ± 1.92	1.98 ± 2.10	0.93 ± 0.80	1.12 ± 0.96	= 0.09	= 0.69
	D_5%_ (Gy)	0.82 ± 0.13	0.86 ± 0.13	0.77 ± 0.06	0.80 ± 0.07	< 0.05	= 0.03
	D_mean_ (Gy)	0.44 ± 0.03	0.45 ± 0.03	0.44 ± 0.02	0.44 ± 0.02	< 0.05	= 0.37
Femur head right	V_30Gy_ (%)	2.03 ± 2.33	2.31 ± 2.42	1.20 ± 0.54	1.38 ± 0.88	= 0.05	= 0.05
	D_5%_ (Gy)	0.87 ± 0.14	0.90 ± 0.14	0.81 ± 0.04	0.83 ± 0.07	= 0.01	= 0.03
	D_mean_ (Gy)	0.45 ± 0.04	0.47 ± 0.04	0.43 ± 0.02	0.45 ± 0.03	< 0.05	= 0.11
Bowel	V_40Gy_ (%)	12.44 ± 2.88	12.19 ± 3.00	12.32 ± 2.85	12.33 ± 3.09	= 0.99	< 0.05
	V_30Gy_ (%)	25.18 ± 3.72	24.79 ± 3.97	24.58 ± 3.84	25.00 ± 3.96	= 0.40	< 0.05
	V_20Gy_ (%)	50.14 ± 3.89	54.43 ± 6.69	46.51 ± 4.26	52.45 ± 6.18	< 0.05	< 0.05
	V_10Gy_ (%)	74.86 ± 4.77	82.38 ± 6.21	70.03 ± 4.93	79.56 ± 5.36	< 0.05	< 0.05
	D_2cm3_ (Gy)	1.67 ± 0.01	1.67 ± 0.01	1.69 ± 0.01	1.69 ± 0.01	= 0.66	< 0.05
	V_40Gy_ (cm^3^)	127.53 ± 48.79	125.12 ± 50.03	126.46 ± 49.01	127.04 ± 52.19	= 0.93	< 0.05
	V_47Gy_ (cm^3^)	0.82 ± 0.83	2.25 ± 2.64	6.96 ± 7.06	7.32 ± 6.28	= 0.67	< 0.05

*p*1 value represents the comparison between S-Adapted plan and S-Scheduled plan; *p2* value represents the comparison between A-Adapted plan and S-Adapted plan

**Table 3:** Dosimetric outcomes for 140 oART fraction of uterine cervical cancer

ROIs	Dosimetric metrics	A-Adapted plan	A-Scheduled plan	S-Adapted plan	S-Scheduled plan	*p*1 value	*p*2 value
CTV-N	V_100%_ (%)	99.83 ± 0.34	99.94 ± 0.09	99.98 ± 0.05	99.95 ± 0.07	< 0.05	< 0.05
CTV-U	V_100%_ (%)	99.35 ± 1.88	98.52 ± 3.80	100.00 ± 0.00	98.93 ± 3.06	< 0.05	< 0.05
CTV-C	V_100%_ (%)	99.30 ± 1.15	98.10 ± 3.31	99.95 ± 0.11	98.36 ± 2.85	< 0.05	< 0.05
PTV-N	V_100%_ (%)	96.56 ± 4.05	97.86 ± 0.95	97.96 ± 1.54	98.16 ± 0.78	< 0.05	< 0.05
PTV-U	V_100%_ (%)	94.20 ± 5.50	93.51 ± 5.26	98.52 ± 1.50	94.27 ± 4.83	< 0.05	< 0.05
PTV-C	V_100%_ (%)	93.01 ± 4.35	93.84 ± 43.67	96.74 ± 2.56	94.20 ± 3.99	< 0.05	< 0.05
Bladder	V_40Gy_ (%)	36.80 ± 6.87	40.55 ± 10.53	42.63 ± 8.58	43.16 ± 9.19	= 0.62	< 0.05
	V_30Gy_ (%)	52.55 ± 6.68	56.47 ± 9.54	59.29 ± 9.70	60.66 ± 8.86	= 0.22	< 0.05
	V_20Gy_ (%)	70.63 ± 7.66	74.20 ± 10.02	76.96 ± 11.13	79.38 ± 10.80	= 0.04	< 0.05
	V_10Gy_ (%)	97.18 ± 2.40	98.44 ± 2.02	98.16 ± 1.85	98.63 ± 1.95	= 0.04	< 0.05
	D_mean_ (Gy)	1.13 ± 0.15	1.23 ± 0.22	1.26 ± 0.19	1.30 ± 0.19	= 0.11	< 0.05
	D_50%_ (Gy)	1.14 ± 0.09	1.20 ± 0.14	1.23 ± 0.13	1.25 ± 0.13	= 0.14	< 0.05
Rectum	V_40Gy_ (%)	53.94 ± 12.24	70.00 ± 12.62	55.08 ± 11.92	70.26 ± 12.58	< 0.05	= 0.43
	V_30Gy_ (%)	70.25 ± 9.60	83.17 ± 8.48	70.83 ± 9.59	82.23 ± 8.62	< 0.05	= 0.62
	V_20Gy_ (%)	82.87 ± 6.10	91.44 ± 5.59	82.95 ± 5.59	89.78 ± 4.46	< 0.05	= 0.91
	V_10Gy_ (%)	92.89 ± 3.88	95.44 ± 3.74	93.24 ± 3.24	95.11 ± 2.79	< 0.05	= 0.41
	D_mean_ (Gy)	1.48 ± 0.21	1.72 ± 0.17	1.50 ± 0.20	1.72 ± 0.15	< 0.05	= 0.50
	D_50%_ (Gy)	1.33 ± 0.13	1.53 ± 0.14	1.34 ± 0.12	1.52 ± 0.13	< 0.05	= 0.48
Bone Marrow	V_10Gy_ (%)	86.77 ± 1.72	87.86 ± 1.75	87.16 ± 1.69	87.30 ± 2.06	= 0.52	= 0.06
	D_90%_ (Gy)	0.31 ± 0.02	0.32 ± 0.02	0.31 ± 0.02	0.31 ± 0.03	= 0.15	= 0.14
	D_mean_ (Gy)	0.94 ± 0.04	0.98 ± 0.04	0.95 ± 0.03	0.98 ± 0.03	< 0.05	= 0.01
Femur head left	V_30Gy_ (%)	2.47 ± 2.19	3.22 ± 2.00	2.71 ± 1.85	3.26 ± 1.79	= 0.01	= 0.34
	D_5%_ (Gy)	0.96 ± 0.09	1.00 ± 0.07	0.97 ± 0.07	1.01 ± 0.07	< 0.05	= 0.12
	D_mean_ (Gy)	0.55 ± 0.06	0.57 ± 0.05	0.55 ± 0.04	0.56 ± 0.03	= 0.04	= 0.51
Femur head right	V_30Gy_ (%)	3.24 ± 2.16	4.40 ± 1.86	3.75 ± 1.80	4.16 ± 1.41	= 0.04	= 0.03
	D_5%_ (Gy)	0.99 ± 0.10	1.05 ± 0.07	1.00 ± 0.07	1.04 ± 0.06	< 0.05	= 0.23
	D_mean_ (Gy)	0.55 ± 0.05	0.57 ± 0.03	0.56 ± 0.03	0.56 ± 0.02	= 0.68	= 0.07
Bowel	V_40Gy_ (%)	34.01 ± 9.12	38.40 ± 10.98	34.04 ± 7.99	39.24 ± 10.92	< 0.05	= 0.97
	V_30Gy_ (%)	49.76 ± 9.09	57.81 ± 10.14	49.98 ± 7.42	58.84 ± 9.86	< 0.05	= 0.83
	V_20Gy_ (%)	62.88 ± 8.13	75.79 ± 9.09	62.79 ± 6.73	75.82 ± 9.49	< 0.05	= 0.92
	V_10Gy_ (%)	78.12 ± 7.48	90.36 ± 8.05	78.27 ± 6.73	89.99 ± 8.34	< 0.05	= 0.86
	D_2cm3_ (cGy)	1.96 ± 0.10	1.98 ± 0.09	1.95 ± 0.08	1.97 ± 0.09	= 0.02	= 0.19
	V_40Gy_ (cm^3^)	247.91 ± 83.42	280.30 ± 103.52	246.44 ± 70.16	284.79 ± 97.21	< 0.05	= 0.87
	V_47Gy_ (cm^3^)	176.98 ± 59.16	206.19 ± 80.72	174.94 ± 46.47	207.83 ± 73.58	< 0.05	= 0.75

*p*1 value represents the comparison between S-Adapted plan and S-Scheduled plan; *p2* value represents the comparison between A-Adapted plan and S-Adapted plan

## Discussion

Currently, a novel artificial intelligence (AI)-driven iCBCT-guided oART has proven feasible in the pelvic region to adapt for daily anatomical variation [22, 23]. de Jong et al. [16] showed that the average time for the adaptive procedure (CBCT2-CBCT1) of neoadjuvant radiotherapy of rectal cancer was 20 min, and Byrne et al. [14] reported that the adaptive time for prostate cancer in the oART emulator was 19 min, which was roughly consistent with our clinical implementation for postoperative and uterine cervical cancer, with average times of 15 min and 18 min, respectively. Daily adaptation was a labour-intensive and time-consuming radiation treatment planning process and needed substantial resources from the department, especially the time for on-site physicians and physicists. Thus, previous studies have mainly focused on weekly adaptation or stereotactic body radiotherapy (SBRT) [16, 23], which could not bring out the full advantages of oART. We anticipated a workflow in which automated segmentation and planning are reviewed online by trained therapists as “adapter” and periodically offline by physicians to ensure that automated segmentation remains appropriate over time, and this work was consistent with the current review process for imaging-guided radiotherapy (IGRT). Our results showed that more than 90% of contouring of influencer structures and CTVs needed no or minor edits based on AI, indicating the feasibility of implementation in postoperative or uterine cervical cancer.

Training of adapters to manage structure editing and identifying key areas for modification are thus crucial. In this study, patients treated with daily oART were prospectively enrolled, and the first iCBCTs were uploaded to the oART simulator to complete the same process without contouring modification. We used metrics such as ASD, centroid deviation, DSC, and 95% HD to assess whether the auto-segmentation performed accurately enough for clinical use and to explore the most deformable region. Our results showed that the DSC was more than 0.75, indicating a high degree of similarity between automatic and manual contours. For cervical cancer treated with radical radiotherapy, the other three metrics reflected that CTV-U had the largest difference, suggesting that the adapter needed to focus on uterus (CTV-U) when modifying the target volume. This was associated with large interfractional variation in the uterus. The uterus movement is greatly affected by bladder filling, which is greater than cervical movement, and the interfractional movement of uterine fundus could be up to 4 cm [24, 25]. This prompted the adapter to focus more on the movement of the uterus when contouring. For postoperative adjuvant radiotherapy, the automatically generated CTV-V_up_ contours had high ASD, centroid deviation, and 95% HD, which was similar to that of the uterus in radical radiotherapy. The CTV-V_up_ includes the vaginal cuff and any visualized paravaginal or retracted parametrial tissue, which is affected by the motion of adjacent organs. When performing daily oART contouring, the auto-contouring system had a higher risk of creating inaccurate contours of the CTVs near the region. These three metrics were also relatively high in CTV-C_up_ compared with CTV-C_down_. For the nodal CTVs, CTV-N1_left_ and CTV-N1_right_ demonstrated relatively low agreement. This may be related to the delineation for daily oART, in which the obturator nodal CTV is carved out of the bladder and does not take interfractional organ motion into account. However, considering that the CTV generated by daily deformation has sufficient coverage to account for motion of the obturator nodal CTV in various states of the bladder, the internal target volume (ITV) was used when contoured in the reference CT. This difference in CTV contouring resulted in the need for daily boundary adjustments in the CTV-N1.

The complex and varied motion of the cervix-uterus target underscores the clinical benefits of oART, and oART is associated with an improvement in dosimetry and the percentage deviation of generalized equivalent uniform dose for the interfractional clinical target volume compared with IGRT [15, 26, 27]. In our study, the targets and OAR dosimetry coverage of A-Adapted plan, A-scheduled plan, S-Adapted plan and S-Scheduled plan on the manually edited contours were assessed. The S-adapted plan improved CTV and PTV coverage compared with the scheduled plan, especially in PTV-V and PTV-U. The scheduled plan was the recalculated reference plan without changing the relevant dose parameters, while the adapted plan was reoptimized based on the current anatomical position. This explains why the adaptive plan resulted in greater improvements for the vaginal cuff and uterine fundus regions where interfractional variation was greater. Thus, correctly adjusting the delineation of the target volume will bring greater clinical benefits. But the results of comparison between A-Adapted plan and S-Adapted plan showed that although the A-Adapted plan met the clinical requirements, it was inferior to the S-Adapted plan. This was in line with our expectations. The reason is that the optimization objectives of A-ART are based on the automatic contours, falling in an incorrect target volume. If the dose is recalculated in the correct modified target volume, there will definitely be a certain gap. Therefore, the results reminded again of the importance of training the adapter to modify the target volume.

The dosimetric superiority of oART was also observed in reducing the dose to OARs. The oART provides an effective way to manage interfraction intestinal peristalsis and bladder-filling movements, as daily adaptive replanning takes into account the patient's anatomy during each fraction with rigorous bladder and rectum preparation, thus explaining the reduced dosimetric sparing in the sessions and reduced toxicity derived from delivering doses high dose to target volume [28, 29]. Earlier studies have demonstrated OAR dosimetric benefits of weekly MR-guided ART that emulated weekly oART significantly reduced the volume of bladder, rectum, bowel, and sigmoid irradiated to all dose levels (V_20Gy_, V_30Gy_, V_40Gy_, V_42.8 Gy_, and V_45Gy_) [30]. Similarly, Liu et al. [31] evaluating the dosimetric benefits of an online adaptive replanning scheme with in-room diagnostic-quality CT scans also showed significant improvements in target coverage and rectal dosimetry. In this experiment, the S-ART plan compared to the S-Scheduled plan achieved significant improvements in bladder rectal and bowel dosimetry with relative reductions from the high-dose region (V_40Gy_) to the low-dose region (V_10Gy_). Moreover, the dosimetric superiority of the adapted plan was also observed in relatively static organs, such as the bone marrow and femoral head. This analysis showed that even using automatically generated contours without manual adjustment improved daily target coverage and OAR dosimetry compared to the scheduled plan. In other words, the uncertainty associated with incorrectly using the system to perform adaptability is still less than the uncertainty associated with approaching the entire process with a single scheduled plan. Moreover, ensuring contour outlines influencers and targets as closely as possible would further amplify this benefit.

Our study had a few limitations. The sample size of this experiment was modelled from all fractions during the full course of oART, and we accurately assessed the intrapatient variability, which represented the scale of motion that occurred in these patients. However, the clinical data were based on a limited cohort of 10 patients, which may underestimate or overestimate the effect of auto-contouring and dosimetry if anatomic outliers were present in these samples. Another potential limitation is the fact that we did not accumulate the dose from the adapted or scheduled plans. We tracked the dose from each fraction independently to accurately assess daily targets and OAR metrics, avoiding compounding errors from an inaccurate dose deformation algorithm. This approach is common in current adaptive studies [32, 33].

## Conclusions

The accuracy of automatic contouring from the Ethos adaptive platform is considered clinically acceptable for cervical cancer, and the resulting daily online adapted plans effectively spare OARs while maintaining a therapeutic dose to the target volume. It is crucial to train adapters, and our results indicated that the uterus, upper vaginal cuff, and low nodal CTV are the areas that need to be focused on.

## Data Availability

All data generated or analysed during this study are included in this published article.

## Abbreviations

PTV: Planning target volume
OARs: Organs at risk
oART: Online adaptive radiotherapy
iCBCT: Iterative cone-beam computed tomography
CTV: Clinical target volume
RTOG: Radiation therapy oncology group
IMRT: Intensity-modulated radiotherapy
ASD: Average surface distance dice
DSC: Dice similarity coefficient
95% HD: 95% Hausdorff distance
SBRT: Stereotactic body radiotherapy
IGRT: Imaging guided radiotherapy
ITV: Internal target volume
